# Oxygen, the Warburg effect, and radiosensitization in malignant epithelial neoplasms

**DOI:** 10.4103/mgr.MEDGASRES-D-25-00253

**Published:** 2026-07-03

**Authors:** André Luiz Sena Guimarães, Andréia Luiza Oliveira Costa

**Affiliations:** Department of Dentistry and Postgraduate Program in Health Sciences, Universidade Estadual de Montes Claros (Unimontes), Montes Claros, Minas Gerais, Brazil

##  

Malignant epithelial neoplasms, particularly oral squamous cell carcinoma (OSCC) and head and neck squamous cell carcinoma, exhibit profound metabolic reprogramming that sustains growth, invasion, and resistance to treatment.^1^,^2^ A hallmark of this adaptation is the Warburg effect, in which tumor cells preferentially utilize glycolysis with lactate production even in the presence of oxygen (**Figure 1**).^3^ This metabolic switch is not a passive consequence of mitochondrial impairment but an active adaptation that provides anabolic intermediates, enhances survival in fluctuating oxygen environments, and promotes aggressive tumor phenotypes.^3^ Hypoxia-inducible factor (HIF) 1α is the central regulator of this reprogramming, modulating genes involved in glucose transport (*GLUT1*), glycolysis (*LDHA*, *HK2*), angiogenesis (*VEGF*), and epithelial–mesenchymal transition-related transcription factors, such as Snail and Twist.^4^ In this way, HIF-1α represents the molecular link between oxygen availability and tumor metabolism, coupling hypoxic stress directly to genetic and metabolic reprogramming.^4^

**Figure 1:**
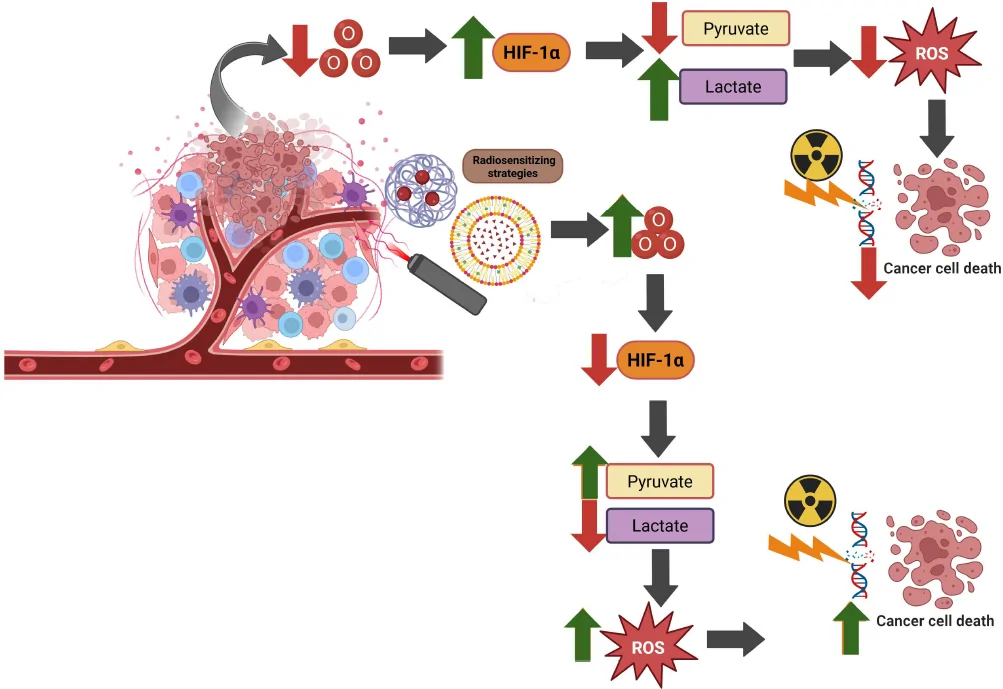
The links between hypoxia, glycolysis, and radiotherapy resistance. Tumor hypoxia decreases local oxygen availability (red arrows), leading to the stabilization and upregulation of HIF-1α. Increased HIF-1α enhances glycolytic flux, elevates lactate production, and reduces pyruvate availability, which together suppress mitochondrial ROS formation. The reduction in ROS limits radiation-induced DNA damage, thereby contributing to radioresistance in neoplastic cells. In contrast, anti-hypoxic and radiosensitizing interventions (e.g., oxygen-releasing biomaterials, metabolic modulators) increase intratumoral oxygenation (green arrows), suppress HIF-1α, restore pyruvate levels, reduce lactate accumulation, and enhance ROS generation following irradiation. The resulting increase in ROS amplifies DNA damage and improves cancer cell killing by radiotherapy. Green arrows indicate upregulation, and red arrows indicate downregulation within each pathway component. Created with BioRender.com. HIF-1α: Hypoxia-inducible factor 1-alpha; ROS: reactive oxygen species.

Historically, hypoxia was considered exclusively a barrier to effective radiotherapy because oxygen is required to stabilize radiation-induced DNA radicals, thereby increasing irreparable DNA damage.^5^ Clinical and experimental observations demonstrated that hypoxic tumor regions correlated with poor radiation responses, giving rise to the concept of the oxygen effect in radiobiology.5 More recent findings suggest that radiotherapy itself can alter tumor metabolism. In patients with OSCC, radiotherapy reduces circulating levels of lactate dehydrogenase, HIF-1α, and miR-210, indicating systemic suppression of glycolytic dominance and hypoxia signaling.^2^ Such findings highlight a bidirectional interaction in which oxygenation status influences radiosensitivity while radiotherapy, in turn, reshapes hypoxia-driven pathways.^2^

The oxygen fixation hypothesis remains fundamental in radiobiology. Ionizing radiation generates transient DNA radicals, and molecular oxygen reacts with these radicals to form peroxyl products that are not amenable to enzymatic repair.^5^ In the absence of oxygen, these radicals are chemically restored, allowing hypoxic cells to resist radiotherapy.^5^ Fractionated radiotherapy schemes rely on reoxygenation, as tumor blood flow and oxygen delivery fluctuate during treatment cycles, enabling partial restoration of oxygen tension and enhanced sensitivity of surviving cells.^5^ Nevertheless, reoxygenation is often incomplete in epithelial tumors, and chronic hypoxia persists as a significant cause of therapeutic failure.^1^

At the molecular level, HIF-1α is stabilized when oxygen levels fall, making it the direct transcriptional sensor of cellular oxygenation.^4^ Under normoxia, prolyl hydroxylase domain enzymes hydroxylate HIF-1α in an oxygen-dependent manner, enabling recognition by the von Hippel–Lindau complex and subsequent ubiquitination and proteasomal degradation.^4^ Under hypoxia, reduced prolyl hydroxylase domain activity prevents hydroxylation, allowing HIF-1α to accumulate, translocate to the nucleus, and heterodimerize with HIF-1β.^4^ The HIF complex then binds hypoxia-response elements, activating genes that enhance glycolysis, angiogenesis, pH regulation, and survival.^4^ Thus, HIF-1α activity is mechanistically dependent on oxygen levels, making it both a biomarker and an effector of hypoxia in tumors.^4^

In OSCC models, hypoxia is associated with reduced E-cadherin expression. It increases migration independently of gene promoter methylation, indicating that epithelial–mesenchymal transition is driven by hypoxia-mediated transcriptional programs rather than irreversible genetic silencing.^6^ Leptin signaling further reinforces these hypoxia adaptations by activating HIF-1α and miR-210, promoting proliferation, invasion, and angiogenesis under reduced oxygen conditions.^7^ These observations exemplify the dual role of HIF-1α: while it supports tumor survival in hypoxia, it simultaneously promotes hallmarks of malignancy that worsen clinical outcomes.^2^,^7^

Natural compounds have been studied as radiosensitizers through their ability to interfere with hypoxia-driven pathways. Gallic acid suppresses HIF-1α and collagen I gene expression while restoring E-cadherin, thereby counteracting epithelial–mesenchymal transition and invasion in OSCC.^8^ More recently, gallic acid was shown to directly inhibit the molecular chaperone heat shock protein 90 alpha family class B member 1 in squamous cell carcinoma, reducing invasion and proliferation while selectively increasing reactive oxygen species (ROS) in malignant but not in normal keratinocytes.^8^ This dual effect, HIF suppression and direct protein targeting, positions gallic acid as a potential radiosensitizer capable of exploiting oxygen’s radical potential.^8^

Metformin, a widely prescribed antidiabetic agent, has also been repurposed as a radiosensitizer in OSCC. It increases pyruvate dehydrogenase activity, reduces HIF-1α stabilization, and decreases mutant p53 accumulation under hypoxic conditions.^1^ In combination with radiotherapy, metformin enhances ROS production, DNA fragmentation, and caspase-3-mediated apoptosis, reversing hypoxia-induced radioresistance.1 Proteomic analyses confirm that metformin modulates glycolytic enzymes, such as glyceraldehyde-3-phosphate dehydrogenase and structural proteins, such as transgelin-2, which are implicated in cellular adaptation to stress and radioresistance.^1^ Thus, metformin exemplifies how targeting metabolic reprogramming can directly sensitize tumors to oxygen-dependent radiation damage.^1^,^2^

Captopril, an angiotensin-converting enzyme inhibitor, represents another pharmacological radiosensitizer. In head and neck squamous cell carcinoma, captopril reduces viability, proliferation, migration, and invasion, while increasing intracellular ROS and apoptosis.^9^ Chorioallantoic membrane assays demonstrate that captopril suppresses tumor-induced angiogenesis, and *in vivo* murine models show that angiotensin-converting enzyme (ACE) inhibition reduces the incidence and severity of carcinogen-induced dysplasia and carcinoma.^9^ Analyses from The Cancer Genome Atlas indicate that downregulation of ACE2 and upregulation of angiotensin II receptor type 1 are associated with a worse prognosis, highlighting that the renin–angiotensin system intersects with hypoxia and angiogenesis in epithelial tumors.^9^ These findings suggest that ACE inhibition could enhance oxygen radical formation and radiosensitivity by suppressing angiogenesis and improving microenvironmental reoxygenation.^9^

Additional factors modulate hypoxia-driven resistance. The prion protein is associated with poor prognosis and lymph node metastasis in head and neck squamous cell carcinoma, correlating with HIF-1α and stress chaperone pathways.^10^ Prion protein overexpression contributes to tumor progression and therapeutic resistance, and its inhibition could indirectly sensitize tumors to oxygen-dependent radiation effects.^10^ Moreover, silver nanoparticles have been investigated for their ability to generate ROS and disrupt mitochondrial respiration, mimicking oxygen radical fixation and providing a potential radiosensitizing effect, though clinical application in epithelial tumors is still under exploration.^10^

These radiosensitizing strategies converge on the oxygen–Warburg axis. By suppressing HIF-1α, reversing glycolytic dominance, and enhancing ROS generation, agents such as metformin, gallic acid, captopril, and silver compounds enable oxygen to function not only as a metabolic substrate but as a therapeutic radical source. Their action can be interpreted within the framework of the 5 Rs (repair, redistribution, reoxygenation, repopulation, and cell rehoming) of radiobiology. They impair DNA repair by overwhelming antioxidant defenses and increasing ROS-mediated lesions.^2^,^3^,^5^ They promote reoxygenation by reducing angiogenic abnormalities and normalizing vascular perfusion.^2^,^4^,^11^ They favor redistribution by modulating hypoxia-induced cell cycle arrest, restoring sensitivity to radiotherapy.^8^ They limit repopulation by suppressing hypoxia-driven stemness and proliferation.^1^,^2^,^4^ Finally, they enhance intrinsic radiosensitivity by augmenting oxygen radical fixation at DNA targets.^4^,^5^

From a translational perspective, oxygen should be considered not merely a limiting factor in radiotherapy but an active therapeutic partner. Future strategies may employ biomaterials such as oxygen microbubbles, perfluorocarbon carriers, photobiomodulation and oxygen-releasing scaffolds to improve local tumor oxygenation during radiotherapy.^5^,^11^ Combining such technologies with pharmacological radiosensitizers could maximize radical fixation and clinical efficacy. Patient stratification using hypoxia signatures, including HIF-1α expression, miR-210 profiles, the ACE/angiotensin II receptor type 1 axis, prion protein status, or heat shock protein 90 alpha family class B member 1 levels, will enable precision medicine approaches that identify individuals most likely to benefit from oxygen-targeted radiosensitization.^2^,^4^,^8^,^9^,^10^ Furthermore, carefully designed clinical trials must integrate metabolic biomarkers, imaging of oxygenation, and optimized fractionation schedules to harness oxygen biology effectively.^4^

In conclusion, malignant epithelial neoplasms illustrate the central role of oxygen and metabolism in determining therapeutic outcomes. The Warburg effect, mediated by HIF-1α, sustains tumor survival in hypoxia but simultaneously creates vulnerabilities that can be exploited through the fixation of oxygen radicals. radiotherapy efficacy depends on oxygen, and pharmacological radiosensitizers such as metformin, gallic acid, and captopril enhance this effect by modulating metabolism, angiogenesis, and redox balance. Oxygen should therefore be reframed not as an obstacle but as a radical source whose potential can be unlocked by combining metabolic therapy, medical gas delivery, and biomaterials. By integrating these approaches, future radiotherapy protocols for epithelial cancers may achieve greater efficacy, personalization, and durability of response.

*This work was supported by grants from the Conselho Nacional de Desenvolvimento Científico e Tecnológico (CNPq – National Council for Scientific and Technological Development, Brazil), the Coordenação de Aperfeiçoamento de Pessoal de Nível Superior (CAPES – Brazilian Federal Agency for Support and Evaluation of Graduate Education), and the Fundação de Amparo à Pesquisa do Estado de Minas Gerais (FAPEMIG – Research Support Foundation of the State of Minas Gerais, Brazil).*

*Dr. Guimarães and Dr. Costa are research fellows of FAPEMIG (Fundação de Amparo à Pesquisa do Estado de Minas Gerais).*

*The authors declare that no Generative AI was used in the preparation of this manuscript.*
